# Dietary Vitamin K2 Isoforms Restore Redox Balance, Organelle Integrity, and Genomic Stability under Streptozotocin-Induced Oxidative Stress in Differentiated SH-SY5Y Cells

**DOI:** 10.1021/acs.jafc.6c03233

**Published:** 2026-10-05

**Authors:** Shruti Shandilya, Janne Ruokolainen

**Affiliations:** Department of Applied Physics, School of Science, Aalto University, Espoo 02150, Finland

**Keywords:** MK-4, MK-7, oxidative stress, DNA damage, mitochondrial dysfunction, Golgi/ER stress, neurite growth

## Abstract

Among the two dietary vitamin K2 isoforms, MK-4 is abundantly found in the brain, and MK-7 is well absorbed and has a longer half-life. Beyond canonical functions, these dietary vitamin K2 vitamers have emerged as bioactive nutrients with potential cytoprotective properties. However, their presence in the brain and their functional relevance in modulating oxidative-stress-associated organelle damage, genomic instability, and neurite integrity remain insufficiently characterized. Streptozotocin led to impaired cellular bioenergetics, followed by organelle damage and genotoxicity. Post-treatment with MK-4 and MK-7 significantly attenuated ROS accumulation, enhanced antioxidant capacity, and preserved mitochondrial functional integrity, as evidenced by improved mitochondrial footprint, reduced mitochondrial ROS, restored ATP levels, and regulated mitophagy. Furthermore, vitamin K2 isoforms mitigated endoplasmic reticulum/Golgi stress, significantly reduced DNA damage, and improved neurite growth. Thus, vitamin K2 isoforms function as multifunctional dietary bioactive compounds with potential relevance for maintaining cell homeostasis during oxidative-stress-associated neuronal dysfunction.

## Graphical abstract

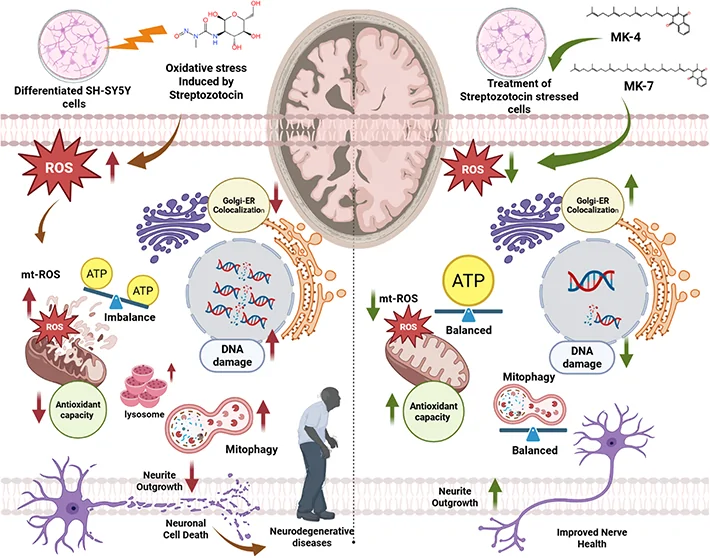

## 1 Introduction

Oxidative stress is a central driver of neuronal dysfunction and progressive neurodegeneration. Excessive generation of reactive oxygen species (ROS) disrupts redox homeostasis, damages DNA, lipids, and proteins, and impairs organelle integrity, ultimately compromising neuronal connectivity and survival.^1,2^ Mitochondria serve as both a primary source and a target of ROS, with oxidative stress-induced mitochondrial dysfunction widely linked to insulin resistance-related neurodegeneration and Alzheimer’s disease (AD)-like pathology.^1,3^ However, neuronal injury under oxidative stress extends beyond mitochondria. Emerging evidence indicates that the endoplasmic reticulum (ER) Golgi axis is highly sensitive to redox imbalance, leading to disrupted protein folding, impaired ER-Golgi trafficking, Golgi fragmentation, and maladaptive stress signaling.^4,5^ Such organelle remodeling is increasingly recognized as a contributor, not merely a consequence, of neurodegenerative progression.

Streptozotocin (STZ), a glucosamine nitrosourea compound with alkylating properties, is commonly employed to model metabolic and oxidative-stress-associated neurotoxicity. In neuronal systems, STZ induces mitochondrial ROS accumulation, bioenergetic impairment, and activation of mitophagy, while also promoting oxidative DNA strand breaks and genomic instability.^3,6^ DNA damage itself can further destabilize Golgi architecture through stress-activated signaling pathways, linking genomic instability to organelle disorganization.^7^ Although mitochondrial dysfunction has been extensively characterized in STZ-based models, coordinated evaluation of ER-Golgi structural integrity, oxidative DNA damage, and neurite degeneration within a unified experimental framework remains limited, particularly in differentiated SH-SY5Y (d-SH-SY5Y) cells, which more closely resemble neuronal phenotypes.

Vitamin K2 (menaquinone) has emerged as a bioactive dietary micronutrient with functions extending beyond its classical role in coagulation. Among its isoforms, menaquinone-4 (MK-4) is selectively enriched in brain tissue, whereas menaquinone-7 (MK-7) exhibits superior bioavailability and longer systemic half-life, enhancing its extrahepatic activity.^8,9^ Nutritional and experimental studies indicate that vitamin K2 exerts antioxidant, antiapoptotic, and mitochondrial protective effects in neuronal systems.^10,11^ In addition, vitamin K participates in sphingolipid metabolism, a pathway critical for membrane organization, growth cone stability, and neurite extension.^8,12^ More recently, vitamin K-based analogues capable of crossing the blood–brain barrier have been shown to enhance neuronal differentiation, underscoring the relevance of menaquinones to neuritogenic signaling.^13^ Importantly, emerging evidence also suggests that vitamin K2 may exert epigenetic regulatory effects relevant to brain health, further expanding its potential role in modulating neuronal resilience.^14^

Despite these advances, prior studies have largely focused on isolated mitochondrial end points or single stress markers, leaving its broader neuroprotective capacity insufficiently defined. To address this gap, we compared MK-4 and MK-7 in differentiated SH-SY5Y cells exposed to streptozotocin-induced oxidative injury using an integrated assessment of mitochondrial ROS, mitophagy, ER/Golgi spatial organization, genomic stability, and neurite architecture. This study provides the first coordinated evidence that nutritionally relevant vitamin K2 isoforms confer multiorganelle and genomic protection under severe oxidative challenge. MK-7 demonstrated better protection than MK-4 in selected end points, underscoring isoform-specific differences that may have implications for dietary strategies aimed at supporting neuronal structural integrity and redox balance in conditions associated with metabolic and neurodegenerative stress.

## 2 Materials and Methods

### 2.1 Reagents

MK-4 and streptozotocin were purchased from Sigma-Aldrich (St. Louis, MO, USA). MK-7 was a kind gift from Gnosis by Lesaffre (Marcq-en-Baroeul, France).

### 2.2 Differentiation of SH-SY5Y Cells

SH-SY5Y cells were provided by Dr. Juan Cruz Landoni, Research Program for Molecular Neurology, University of Helsinki. Cells were maintained in DMEM (Thermo Fisher Scientific, USA) supplemented with 10% FBS (Thermo Fisher Scientific, USA), streptomycin (50 ug/mL)/penicillin (50 U/mL) (Thermo Fisher Scientific, USA) at 37 °C in a humidified incubator with a supply of 5% CO_2_. Differentiation of SH-SY5Y cells into more mature neuron-like cells was achieved by culturing them in gradual serum starvation as described previously^15,16^ with few modifications. Briefly, SH-SY5Y cells were plated in 96- and 6-well plates according to their respective densities and experiments and were maintained in differentiation media 1 (Table 1) containing low serum and RA at days 1, 3, and 5. At day 7, cells were further cultured in differentiation media 2 (Table 1). From the start of differentiation, media was replaced by fresh medium every 48 h. The timeline of the differentiation process is shown in Figure 1. At day 15, differentiation of SH-SY5Y cells was recognized by long extended branch processes expressing markers of mature neurons: microtubule-associated protein 2 (MAP2) (Thermo Fisher Scientific, USA, 1:100) and neuronal nuclei (NeuN) (Sigma-Aldrich, USA, 1:100) by immunocytochemistry. Briefly, after differentiation, cells were washed in warm PBS, fixed in 4% paraformaldehyde, and then permeabilized with 0.25% Triton X-100 and blocked with a blocking buffer containing 10% goat serum. Cells were then incubated with primary antibodies overnight, with subsequent incubation with Alexa fluor (488)-conjugated secondary antibodies (Thermo Fisher Scientific, USA). Fluorescence was visualized using an Axio Observer Z1 microscope (Carl Zeiss, Jena, Germany) with a 20× objective lens (λ_ex_ = 490 nm, λ_em_ = 525 nm).

**Figure 1:**
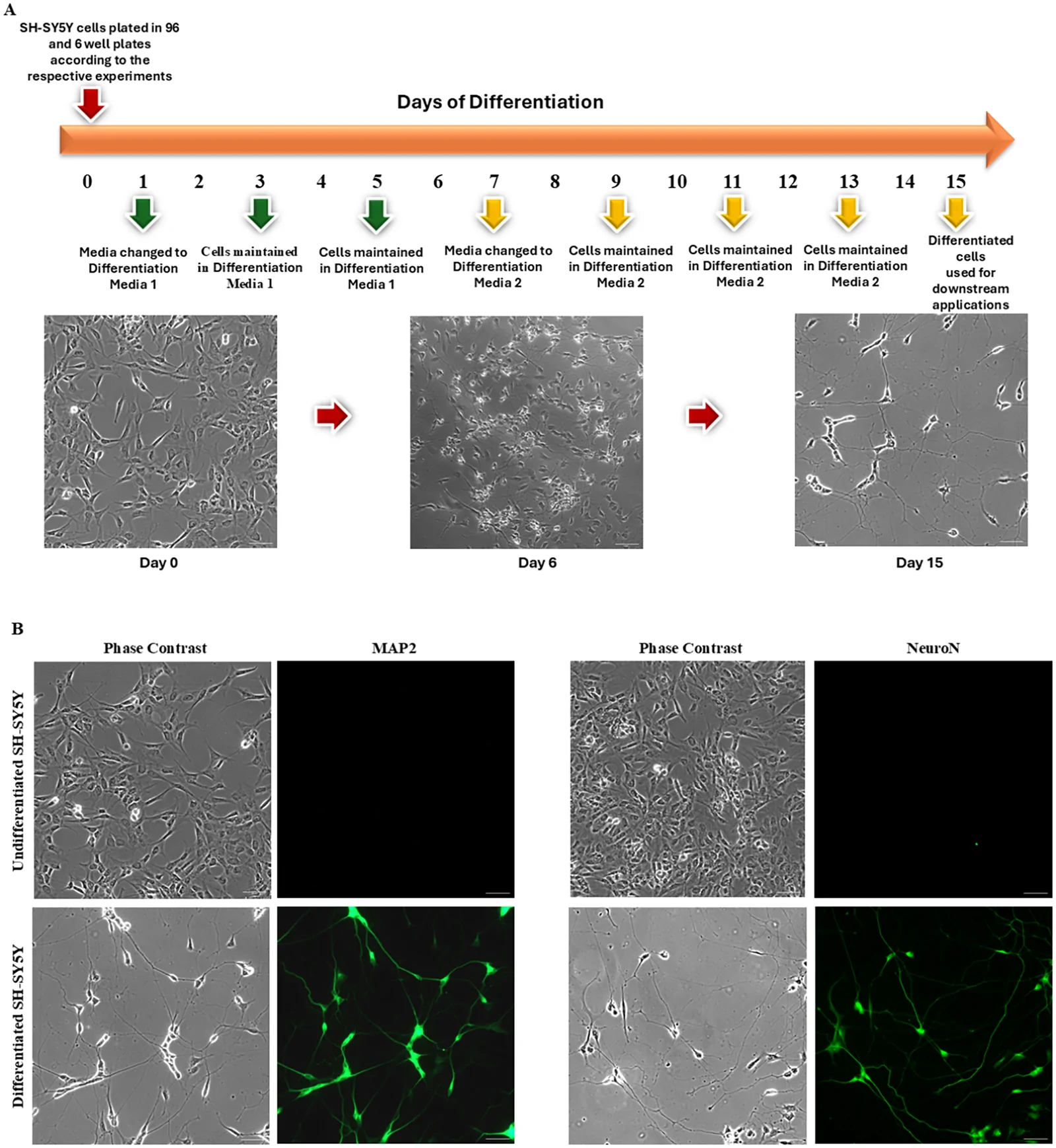
Differentiation of SH-SY5Y cell line. (A) Timeline of the differentiation procedure from day 0 to day 15, which consists of 9 steps to obtain differentiated neuron-like cells used further for downstream applications. Phase-contrast micrographs show that at day 0, undifferentiated SH-SY5Y cells possess a phenotype with few projections, while at day 6, slightly longer neurite projections are observed, and finally, at day 15, differentiated SH-SY5Y cells possess elongated neurite projections (scale bar: 100 μm). (B) Fluorescence microscopy images show the expression of neuronal differentiation markers MAP2 and NeuN (green-AF488) at day 15 of differentiation, which was not found to be expressed in undifferentiated cells. Corresponding phase-contrast images of each immunofluorescence channel are shown on the left (scale bar: 100 μm).

**Table 1:** Components of Differentiation Media

**differentiation media 1**	**concentration**
DMEM (Thermo Fisher Scientific, USA)	
FBS (Thermo Fisher Scientific, USA)	1.5 %
penicillin/streptomycin (Thermo Fisher Scientific, USA)	1 %
retinoic acid (Sigma-Aldrich, USA)	10 μM (25 mM stock)
differentiation media 2	
Neurobasal^TM^ medium (Thermo Fisher Scientific, USA)	
B-27^TM^ supplement (Thermo Fisher Scientific, USA)	1× (50× stock)
KCl (Sigma-Aldrich, USA)	20 mM (1 M Stock)
penicillin/streptomycin (Thermo Fisher Scientific, USA)	1×
GlutaMax^TM^ supplement (Thermo Fisher Scientific, USA)	2 mM (100× stock)
BDNF (peprotech proteins, Thermo Fisher Scientific, USA)	50 ng/mL (10 μg/mL)
db-cAMP (Sigma-Aldrich, USA)	2 mM (1 M stock)
RA (Sigma-Aldrich, USA)	10 μM (25 mM stock)

### 2.3 Experimental Design

To study organelle-protective and antioxidative effect of dietary vitamin K2 isoforms on neuronal toxicity induced by STZ, d-SH-SY5Y cells were exposed to increasing concentrations of streptozotocin (0.1, 0.5, 1, and 1.5 mM) for 24 h, followed by the post-treatment of MK-4 and MK-7 at a concentration of 50 μM for 24 h. To investigate the role of dietary vitamin K2 isoforms at the highest concentration of streptozotocin, streptozotocin at 1.5 mM and MK-4 and MK-7 at 50 μM were investigated in experiments involving organelle stress assessment, genotoxicity, and neurite outgrowth analysis. The controls used in this study include untreated differentiated controls, STZ-only injury controls, and vitamin K2 (MK-4 and MK-7) only treatment groups where applicable. Experiments were carried out in triplicate and quadruplicate. All data presented are blank corrected.

### 2.4 Cell Viability and Toxicity

The cytotoxic effects of STZ towards d-SH-SY5Y cells and the cytoprotective effects of post-treatment of MK-4 and MK-7 were determined using 3-(4,5-dimethylthiazol-2-thiazolyl)-2,5 diphenyl tetrazolium bromide (MTT; Sigma-Aldrich, USA).^10^ Briefly, d-SH-SY5Y cells were plated at a density of 1.5 × 10^4^ cells/well in 96-well plates. After treatment, for the MTT assay, the media was replaced with medium containing assay-specific probe MTT (5 mg/mL) and incubated for 3 h at 37 °C. The formazan crystals formed by viable cells were solubilized in DMSO. Absorbance was measured at 570 nm using a microplate reader (Cytation 3, BioTek Instruments, USA). Further, the release of LDH caused by damage to the plasma membrane was quantified using the CYQUANT LDH Cytotoxicity Assay Kit (Invitrogen, Thermo Fisher Scientific, USA) following the manufacturer’s instructions. LDH release was measured spectrophotometrically at 490 and 680 nm using a microplate reader (Cytation 3, BioTek Instruments, USA).^10^ Total LDH release is the sum of intracellular and extracellular LDH activity and is expressed as a percentage of the total value.

### 2.5 Cellular Metabolic and Redox Status

#### 2.5.1 Intracellular ATP Quantification

Changes in intracellular ATP levels in d-SH-SY5Y cells during metabolic stress induced by STZ and post-treatment of MK-4 and MK-7 were quantified using a luminescence-based ATP Determination Kit (Invitrogen, Thermo Fisher Scientific, USA). Briefly, 1 × 10^5^ cells/well were plated in 6-well plates for differentiation. After differentiation and treatment, cells were treated and processed as per the manufacturer’s protocol. ATP luminescence signal was measured using a microplate reader (Cytation 3, BioTek Instruments, USA). ATP signals were background-corrected and normalized to the protein content of the cells.

#### 2.5.2 Intracellular ROS Measurement

Intracellular ROS production was assayed using 2,7-dichlorofluorescein diacetate (DCFH-DA) (Sigma-Aldrich, USA).^10^ Immediately after differentiation and treatment, the medium was removed from the wells and replaced with DCF-DA (25 μM) in 0.5 mL of HBSS, followed by incubation for 30 min at 37 °C. Fluorescence was then measured at 485 nm excitation/535 nm emission wavelengths using a Cytation Reader 3 (BioTek Instruments, USA).

#### 2.5.3 Antioxidant Capacity

The antioxidant capacity of d-SH-SY5Y cells in response to STZ treatment followed by post-treatment with MK-4 and MK-7 was assessed using the Antioxidant Assay Kit (Sigma-Aldrich, USA). SH-SY5Y cells were differentiated and treated in 6-well plates at a density of 1 × 10^5^ cells/well. Antioxidant capacity was then determined using the manufacturer’s protocol. Absorbance was measured at 405 nm using a plate reader (Cytation 3, BioTek Instruments, USA). Antioxidant concentrations were expressed as Trolox equivalents (mM).

### 2.6 DNA Damage Assay

To assess DNA strand breaks at the single-cell level, a comet assay was performed using the Comet SCGE assay kit (Enzo Life Sciences, USA) and as described previously.^17^ 1 × 10^5^ cells/well were differentiated and subsequently treated in 6-well plates. After treatment, the comet assay was performed as per the manufacturer’s protocol. Briefly, cells were harvested and embedded in low-melting-point agarose on microscope slides. After agarose solidification, slides were incubated in cold lysis buffer for 1 h to remove cellular membranes and proteins. DNA was then allowed to unwind under alkaline conditions (pH > 13) for 30 min in the dark, followed by washing slides in 1x TBE buffer for 5 min 2×. Horizontal gel electrophoresis was performed at 25 V/300 mA for 30 min. Slides were neutralized, subsequently dipped into 70% ethanol for 5 min, air-dried, and stained with CYGREEN DNA-binding fluorescent dye for 30 min in the dark. Images from fluorescently stained samples were captured using an FITC filter (λ_ex_ = 489 nm, λ_em_ = 515 nm) through Nikon Plan Apo (10×) air objectives using a Nikon Eclipse-E upright microscope (Nikon, Tokyo, Japan). For each treatment condition, at least 100 comets were randomly selected and analyzed. DNA damage was quantified using CometScore 2.0 software (http://rexhoover.com/index.php?id=cometscore) and expressed as % tail DNA and Olive tail moment.

### 2.7 Image Processing

To improve contrast and out-of-focus blur for accurate quantification of mitochondrial stress parameters like mitophagy, mitochondrial footprint, and Golgi/ER stress, image deconvolution was performed using NIH ImageJ software as described previously.^18^ Briefly, a theoretical 3D point spread function (PSF) was generated using the Diffraction PSF 3D plugin, and images were subsequently deconvolved using the Iterative Deconvolution 3D plugin. Identical deconvolution parameters were applied across all experimental groups, and the deconvolved images were used for visualization and a subsequent quantitative analysis.

### 2.8 Mitochondrial Stress Signature

#### 2.8.1 MitoSOX/Mitochondria Signal Ratio (mt-ROS)

MitoSOX-to-mitochondria signal ratio was used as an index of mitochondrial ROS levels normalized to mitochondrial content.^19^ Generation of mt-ROS was determined by using the fluorescence probe MitoSOX Red (Sigma-Aldrich, USA), and mitochondria were visualized using Biotracker Green Dye (Sigma-Aldrich, USA). 5 × 10^3^ cells/well were plated in 8-well chamber slides and were differentiated, followed by treatment with STZ and post-treatment with MK-4 and MK-7. After treatment, the media was replaced with HBSS containing MitoSOX Red (500 nM) and Biotracker Green (200 nM), and the cells were incubated for 30 min. After staining, cells were rinsed 3 times with pre-warmed HBSS and immediately imaged in live cell conditions. Fluorescence was visualized by an Axio Observer Z1 microscope (Carl Zeiss, Jena, Germany) with a 20× objective lens (MitoSOX Red: λ_em_ = 580 nm; Biotracker Green: λ_em_ = 523 nm). Fluorescence images were acquired using identical exposure settings across all conditions. The MitoSOX fluorescence intensity was quantified within mitochondria-positive regions of interest (ROIs) and normalized to the corresponding mitochondrial marker intensity to obtain the MitoSOX/mitochondria signal ratio. Data were quantified utilizing 90–100 ROIs from each treatment group.

#### 2.8.2 Mitophagy

Mitophagy was evaluated by the degree of colocalization between mitochondria and lysosomes.^20^ To ascertain this, 5 × 10^3^ cells/well were plated in 8-well chamber slides and were differentiated, followed by treatment with STZ and post-treatment with MK-4 and MK-7. After treatment, the media was replaced with HBSS containing Lysotracker Deep Red (Thermo Fisher Scientific, USA) (200 nM) and Biotracker Green (Sigma-Aldrich, USA) (200 nM) and was incubated for 30 min. After staining, cells were rinsed thrice with pre-warmed HBSS and immediately imaged in live cell conditions. Two-channel fluorescence was visualized by an Axio Observer Z1 microscope (Carl Zeiss, Jena, Germany) with a 20× objective lens (Lysotracker Deep Red λ_em_ = 668 nm; Biotracker Green: λ_em_ = 523 nm). Fluorescence images were acquired using identical exposure settings across all conditions. Mean green fluorescence intensity of mitochondria and red fluorescence intensity of lysosomes were quantified by NIH ImageJ software. Colocalization between mitochondria and lysosomes was determined by the merged tangerine color in fluorescence images and was quantified through the JACoP plugin^21^ of the NIH ImageJ software. M2 Manders’ colocalization coefficients (MCC) of mitochondria overlapping with the lysosomes are represented utilizing 90–100 ROIs from each treatment group.

#### 2.8.3 Mitochondrial Footprint

Changes in mitochondrial morphology in response to treatment with STZ and post-treatment with MK-4 and MK-7 were measured by fluorescence microscopy using Biotracker Green Dye (Sigma-Aldrich, USA). Briefly, 5 × 10^3^ cells/well were plated in 8-well chamber slides and were differentiated. After treatment, cells were stained with Biotracker Green (200 nM) and incubated for 30 min. After being fixed with 4% paraformaldehyde, fluorescence images were captured by an Axio Observer Z1 microscope (Carl Zeiss, Jena, Germany) with a 20× objective lens (Biotracker Green: λ_em_ = 523 nm). To quantitatively assess cellular mitochondrial networks, the original images were pre-processed with ImageJ software using “unsharp mask,” “enhance contrast,” “median,” “binarize,” “skeletonize,” and “analyze skeleton” functions. Further, to simplify the analysis, a semiautomated ImageJ plugin, MiNA, was used to determine mitochondrial footprint in zoomed images.^22^ The mitochondrial footprint, defined as the total mitochondrial signal area after background subtraction, was selected as a representative parameter of mitochondrial structural integrity. Data were quantified utilizing at least 90–100 ROIs from each treatment group.

### 2.9 Determination of Golgi/ER Stress

Golgi/ER stress and colocalization representing Golgi/ER spatial organization were visualized by fluorescence microscopy using Cytopainter-Golgi/ER staining kit (Abcam, Cambridge, UK).^23^ 5 × 10^3^ cells/well were plated in 8-well chamber slides and were differentiated. Followed by the treatment, cells were stained with Cytopainter-Golgi/ER stain according to the manufacturer’s instructions. Two-channel fluorescence was visualized by an Axio Observer Z1 microscope (Carl Zeiss, Jena, Germany) with a 40× objective lens. Green fluorescence represents the Golgi (λ_em_ = 519–525 nm), red fluorescence represents the ER (λ_em_= 610–615 nm), and colocalization between the two was visualized as bright yellow. Images were background subtracted, and individual cells were defined as ROIs. Data were quantified utilizing 90–100 ROIs from each treatment group. ER and Golgi signals were quantified as mean fluorescence intensity using ImageJ software. Colocalization between ER and Golgi was assessed in ImageJ/Fiji using the JACoP plugin^21^ and reported as standard colocalization metrics, Manders’ colocalization coefficients (MCC).

### 2.10 Neurite Outgrowth Analysis

Neurite growth represents the nerve cell connectivity and normal functioning of the nervous system.^24^ Change in neurite growth in response to STZ treatment, followed by the post-treatment of MK-4 and MK-7, was studied. Briefly, SH-SY5Y cells were differentiated and treated in 6-well plates at a density of 1 × 10^5^ cells/well. After differentiation, treatment, and fixation with 4% paraformaldehyde, phase-contrast images were acquired with an Axio Observer Z1 microscope (Carl Zeiss, Jena, Germany) with a 20× objective lens. For quantitative analysis, at least 10 micrographs from each treatment group were randomly selected. In the determined fields, all neurites were measured using the NeuronJ plugin of ImageJ software.^25^ For this analysis, the number of cell bodies or the cell body area was not evaluated, and only the neurite length was quantified. Neurite length is expressed in μm.

### 2.11 Statistical Analysis

All data analyzed are presented as mean ± SEM. GraphPad Prism software (version 10.6.1) (GraphPad Software, San Diego, CA) was used for the statistical analysis. A *t* test was used to analyze the differentiation marker expression between the two groups (SH-SY5Y and d-SH-SY5Y), and differences with a *p ≤ 0.05 were considered as significant. Whereas, more than two treatment groups were analyzed using one-way analysis of variance (ANOVA), followed by Tukey’s post-hoc test. Differences with *p ≤ 0.05, **p ≤ 0.01, ***p ≤ 0.001, and ****p ≤ 0.0001 were considered statistically significant.

## 3 Results and Discussion

### 3.1 Differentiation of SH-SY5Y Cells

SH-SY5Y neuroblastoma cell line has a stable karyotype consisting of 47 chromosomes, which can be differentiated from a neuroblast-like state into a mature neuron-like cell population, thus making it a consistent and reproducible human neuronal *in vitro* cell testing model for different neurobiological experiments.^26,27^ Toward this approach, utilizing available protocols^15,16^ with minor modifications, a stepwise differentiation protocol (Figure 1A) was employed to generate a mature neuron-like population. At day 0, cells possess an undifferentiated phenotype with very few and short processes, which can rapidly proliferate and appear to be non-polarized, expressing markers of immature neurons.^28^ Retinoic acid (RA)-based differentiation under low serum conditions is known to initiate neuronal commitment and neurite outgrowth through transcriptional regulation of neuronal genes and cytoskeletal remodeling.^27,29^ Consistent with these reports, exposure to low serum medium with RA from day 1 to 6 leads to an outgrowth of neurites as observed in phase-contrast images (Figure 1A). Further, with subsequent exposure to neurobasal medium containing extracellular matrix proteins and BDNF, cells were able to develop long neurites as observed at day 15 of differentiation (Figure 1A). BDNF is known to be a key regulator of neuronal survival and neurite outgrowth, acting through TrkB-mediated signaling pathways to support neuronal structural and functional maturation.^30^ Further, it has also been observed that removal of BDNF resulted in cellular apoptosis,^29^ suggesting that, similar to mature human neurons, survival of differentiated SH-SY5Y also depends on trophic factors. Previous reports demonstrated that fully differentiated SH-SY5Y cells express a variety of different markers of mature neurons, including GAP-43, NeuN, MAP, SYN, NSE, and SV2.^16,26,27,31^ Similarly, in this study, immunocytochemistry revealed that cells at day 15 expressed mature neuronal marker proteins MAP2 and NeuN (Figure 1B), which were not observed in undifferentiated cells. Thus, the coordinated increase in neurite outgrowth and neuronal marker expression confirms the successful generation of a differentiated SH-SY5Y (d-SH-SY5Y) neuronal model, in agreement with previous studies.^15,16^ Accordingly, oxidative stress-induced d-SH-SY5Y cells were employed as a cellular model to examine the neuroprotective potential of dietary vitamin K2 isoforms. This experimental framework allows assessment of bioactive food components in a neuron-like context that better reflects the cellular vulnerability observed in neurodegenerative conditions.

### 3.2 MK-4 and MK-7 Improve Cell Viability and Reduce Toxicity in d-SH-SY5Y Cells

Streptozotocin is a naturally occurring, highly toxic glucosamine nitrosourea compound, which is well established to develop experimental models of diabetic neuropathy and sporadic Alzheimer’s disease *in vivo*, as it causes insulin resistance, oxidative damage, and neurodegeneration.^32,33^ *In vitro*, STZ exerts dose-dependent cytotoxic effects and disrupts cellular metabolism in neural cell systems, making it a useful tool to study neuronal injury and protective interventions.^10,34^ In this study, the cytotoxic effect of STZ on the viability of d-SH-SY5Y cells was assessed using the MTT and LDH assays. The MTT assay assesses mitochondrial metabolic activity through reduction of the tetrazolium salt, MTT, whereas the LDH assay measures plasma membrane damage by quantifying the release of cytoplasmic lactate dehydrogenase.^35^ STZ treatment (0.1–1.5 mM) for 24 h significantly reduced MTT signal and increased LDH release compared with non-treated control cells within each experimental set, indicating reduced mitochondrial metabolic activity and loss of membrane integrity. Among the tested concentrations, 1.5 mM STZ produced the maximum and significant reduction in d-SH-SY5Y cell viability (Figure 2A,B). Previous studies have demonstrated that streptozotocin exerts concentration-dependent cytotoxic effects that differ between undifferentiated and differentiated SH-SY5Y cells, reflecting their distinct metabolic and neuronal states.^34^ While vitamin K2 has been reported to exhibit non-cytotoxic effects and a favorable safety profile in previous studies.^36^ Similarly, treatment with vitamin K2 isoforms MK-4 and MK-7 (50 μM) alone did not induce cytotoxicity in d-SH-SY5Y cells when compared with untreated controls. Notably, post-treatment of STZ-stressed cells with MK-4 and MK-7 (50 μM) for 24 h significantly reduced LDH release and increased MTT signal compared with the corresponding STZ-only groups within each experimental set (Figure 2A,B). Similar neuroprotective potential of vitamin K2 has been demonstrated across diverse cellular models. In undifferentiated SH-SY5Y cells, post-treatment with MK-4 significantly reduced cytotoxicity induced by streptozotocin and menadione.^10^ Also, MK-7 isolated from natto attenuated 6-hydroxydopamine-induced cytotoxicity in SH-SY5Y cells.^11^ In differentiated rat pheochromocytoma PC12 cells, pre-treatment with vitamin K2 reduced amyloid-β (Aβ_1–42_) peptide- and hydrogen peroxide-induced toxicity.^37^ Vitamin K2 was also shown to mitigate rotenone-induced neurotoxicity in SH-SY5Y cells via conditioned medium from BV2 microglial cells treated with rotenone and MK-4, thereby reducing microglia-mediated neuronal cell death.^38^ Furthermore, in astroglioma C6 cells, vitamin K2 conferred dose-dependent protection against Aβ-mediated cytotoxicity.^39^ Beyond its neuroprotective actions, vitamin K2 has been shown to protect proximal renal tubular cells from BSO-induced cell death^40^ and has been shown to restore osteoblast function in both *in vitro* and *in vivo* models.^41^ In our study, both MK-4 and MK-7 improved membrane integrity by reducing STZ-induced LDH release compared with their corresponding STZ-only groups within each experimental set (Figure 2B). MK-7 appeared to produce a greater reduction in LDH release in selected conditions. This difference may be related, at least in part, to isoform-specific properties such as the longer isoprenoid side chain of MK-7 and its potential influence on cellular retention or membrane association^9,42^ ;^9,42^ however, intracellular MK-4 and MK-7 levels were not directly measured in this study. Collectively, these findings demonstrate that MK-4 and MK-7 attenuated STZ-induced membrane damage and effectively restored cell viability, supporting their cytoprotective potential under STZ-induced stress conditions.

**Figure 2:**
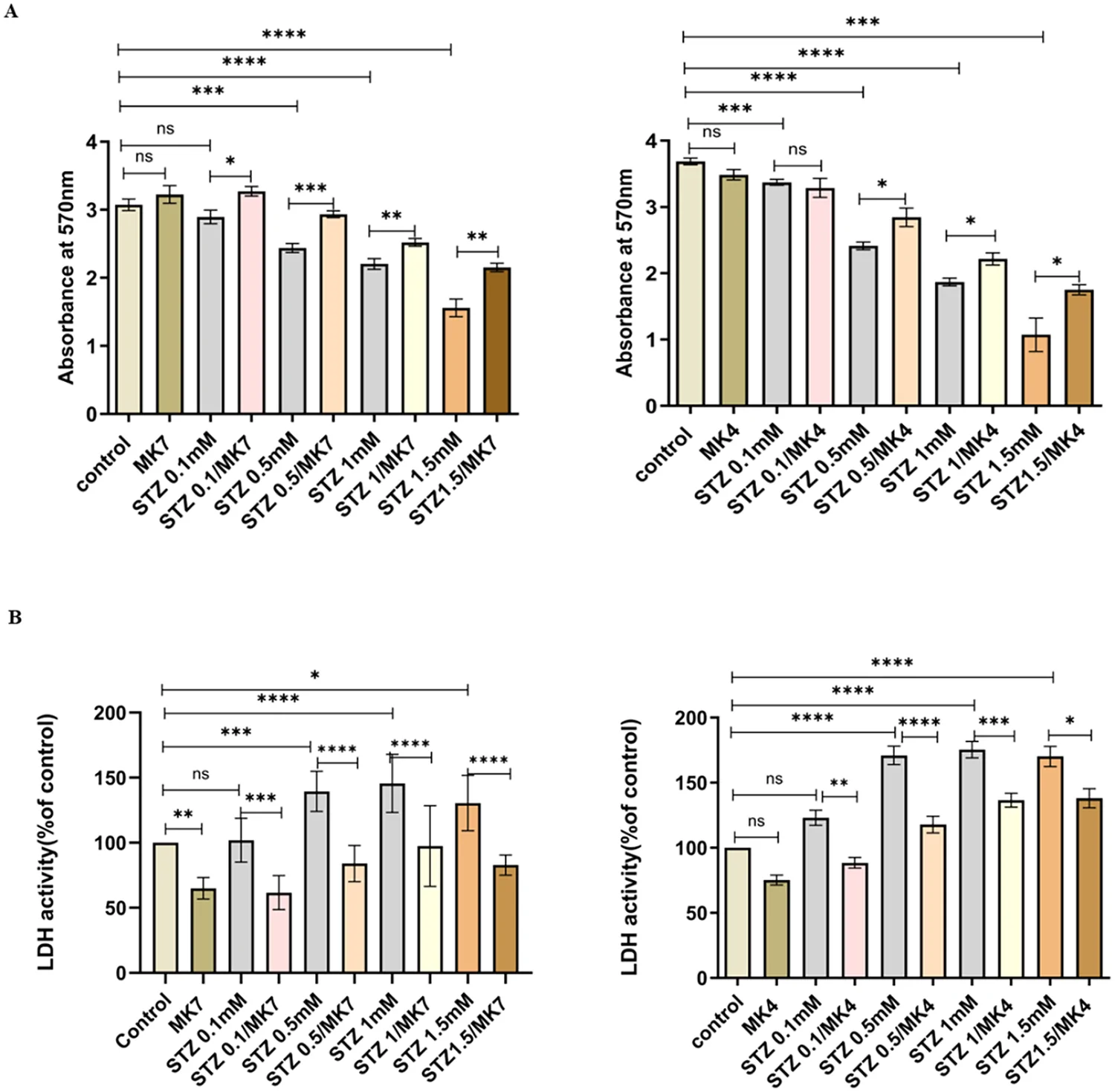
Effect of MK-4 and MK-7 on cell viability and toxicity in d-SH-SY5Y cells exposed to STZ. (A) Cell viability was assessed using the MTT assay (*n* = 3). (B) Cytotoxicity was evaluated by measuring lactate dehydrogenase (LDH) release in culture supernatants (*n* = 3). Data are presented as mean ± SEM. Statistical significance was determined by one-way ANOVA followed by Tukey’s post-hoc test; differences with *p*-values ≤0.05 were designated as significant. Brackets indicate the exact between-group comparisons, including untreated control versus STZ-only groups, untreated control versus MK-4/MK-7-only groups, and STZ-only groups versus corresponding MK-4- or MK-7-treated groups; ns, not significant; *p < 0.05, **p < 0.01, and ***p < 0.001 denote significant differences compared with the control and between the treatment groups. MK-7, menaquinone-7; MK-4, menaquinone-4; STZ, streptozotocin; STZ/MK-4, post-treatment of STZ-stressed cells with MK-4; STZ/MK-7, post-treatment of STZ-stressed cells with MK-7.

### 3.3 MK-4 and MK-7 Regain Cellular Homeostasis and Improve Cell Health

Impaired ATP production and oxidative damage, which are caused by a serious imbalance in ROS generation and antioxidative defense mechanisms of the cell, are found to be intimately correlated in AD and aMCI brain.^43^ STZ is well established to induce oxidative stress through excessive ROS generation and depletion of antioxidant defenses in neuronal models.^3^ To ameliorate the oxidative stress induced by streptozotocin in d-SH-SY5Y cells, the role of exogenous dietary vitamin K2 isoforms was studied. It was observed that STZ treatment significantly disrupted cellular homeostasis within each experimental set, as evidenced by a marked increase in ROS generation in STZ treatment (0.1–0.5 mM), followed by the subsequent decrease at the higher STZ concentration (1–1.5 mM) in comparison to that of control untreated cells, as shown in Figure 3A. The decrease in ROS generation at high STZ concentration was due to reduced viability and metabolic activity of cells, rather than diminished oxidative stress per se. Whereas post-treatment with MK-4 and MK-7 significantly attenuated ROS generation even at the highest concentration of STZ, confirming the antioxidative role of vitamin K2 isoforms (Figure 3A). STZ significantly reduced the antioxidant capacity of cells in a dose-dependent manner when measured to TROLOX equivalents, which is consistent with the significant reduction in antioxidant enzyme activities like SOD and CAT, and non-enzymatic antioxidants like reduced GSH in pancreatic, liver, and kidney tissues.^44,45^ While treatment of d-SH-SY5Y cells with MK-7 and MK-4 alone significantly increased the cellular antioxidant capacity compared with untreated controls, suggesting the enhanced antioxidant defense mechanism under basal conditions. Also, post-treatment of STZ-stressed cells with MK-7 and MK-4 resulted in a significant increase in the antioxidant capacity, demonstrating recovery of the cellular redox defense system (Figure 3B). This effect would be mediated through direct radical scavenging and preservation of mitochondrial function, as previously described for vitamin K2 in neuronal systems.^8,10^ Similarly, inhibition of oxidative stress by vitamin K2 protected developing oligodendrocytes and immature fetal cortical neurons from glutathione depletion-induced cell death.^46^ Vitamin K2 was also found to confer protection against oxidative stress-induced neural injury by suppressing 12-lipoxygenase activation and reducing ROS accumulation during arachidonic acid-mediated damage in developing oligodendrocytes, leading to improved cell survival.^47^ Further, vitamin K2 was found to reduce oxidative stress and neuroinflammation while reducing acetylcholinesterase in prolonged diabetic-induced damage in hippocampal neurons,^48^ followed by its antioxidative defense mechanism observed against H_2_O_2_ ^37^ and Aβ_(1–42)_.^39^ Excessive ROS generation causes mitochondrial dysfunction and impairs its ability to maintain normal levels of ATP.^49^ Studies done in previous years have reported that vitamin K2 maintains normal ATP production by acting as a mitochondrial electron carrier and is required within the electron transport chain to protect impaired mitochondrial function.^50,51^ Our results also showed that treatment of d-SH-SY5Y cells with MK-7 alone significantly elevated ATP levels in comparison to MK-4 and untreated control cells, suggesting that it enhanced the mitochondrial or metabolic efficiency even under normal conditions (Figure 3C). In contrast, within each experimental set, treatment of d-SH-SY5Y cells with streptozotocin resulted in a significant disruption of cellular homeostasis, as evidenced by a marked reduction in intracellular ATP levels at concentrations of 0.1 and 0.5 mM, consistent with its known ability to impair mitochondrial function and induce metabolic stress in neuronal cells.^3,52^ Interestingly, it has been observed that STZ at a concentration of 1 mM resulted in a gradual increase in ATP, followed by a very sharp increase at the highest critical concentration of STZ, 1.5 mM, as shown in Figure 3C, which may reflect a stress-induced metabolic imbalance or compensatory response preceding loss of neuronal cell viability. This phenomenon was also observed in intact HeLa, PC12, and U937 cells, where cytosolic ATP within cells undergoing apoptosis is maintained at a level higher than in control cells, even as caspase activation and DNA laddering are occurring, during the final stages of cell death, suggesting that elevation of cytosolic ATP level is requisite to apoptosis.^53^ Similarly, glucose deprivation caused by STZ led to altered energy requirements and mitochondrial activity, leading to neurodegeneration.^54^ Also, glucose deficiency in MCF-7 cells directly elicited necrosis depending on ATP generation instead of ATP depletion.^55^ After seeing a sharp rise in cytosolic ATP levels in STZ-treated d-SH-SY5Y cells, post-treatment of STZ-stressed cells with MK-4 and MK-7 for 24 h resulted in normalized ATP levels to values comparable with control and vitamin K2 alone-treated cells within each experimental set, indicating restoration of energy homeostasis (Figure 3C). Similarly, MK-7 was found to reduce hypoxia-induced oxidative stress in astrocytes by balancing ROS production and optimizing ATP generation.^56^ Notably, VK2 has been found to facilitate ATP production by working as a mitochondrial electron carrier in Drosophila and rescues mitochondrial dysfunction caused by PINK1 mutations.^51^ Recently, it has been reported that VK2 also restored the reduced ATP levels caused by mitochondrial abnormalities during fertilization in *Caenorhabditis elegans* embryos.^57^ In contrast, it has been reported that VK2 as an electron carrier is only found in Drosophila but cannot substitute for COQ10 as an electron carrier in the mitochondrial respiratory chain of HEK293, HeLa cells, and human fibroblast cells.^58^ Inclusively, results from our study demonstrated that MK-4 and MK-7 effectively restore cellular homeostasis under STZ-induced stress by normalizing energy metabolism, enhancing antioxidant defenses, and reducing oxidative stress. The more pronounced effects observed with MK-7 may reflect isoform-specific differences that are consistent with reports of greater bioavailability and intracellular persistence compared with MK-4.^9,59^

**Figure 3:**
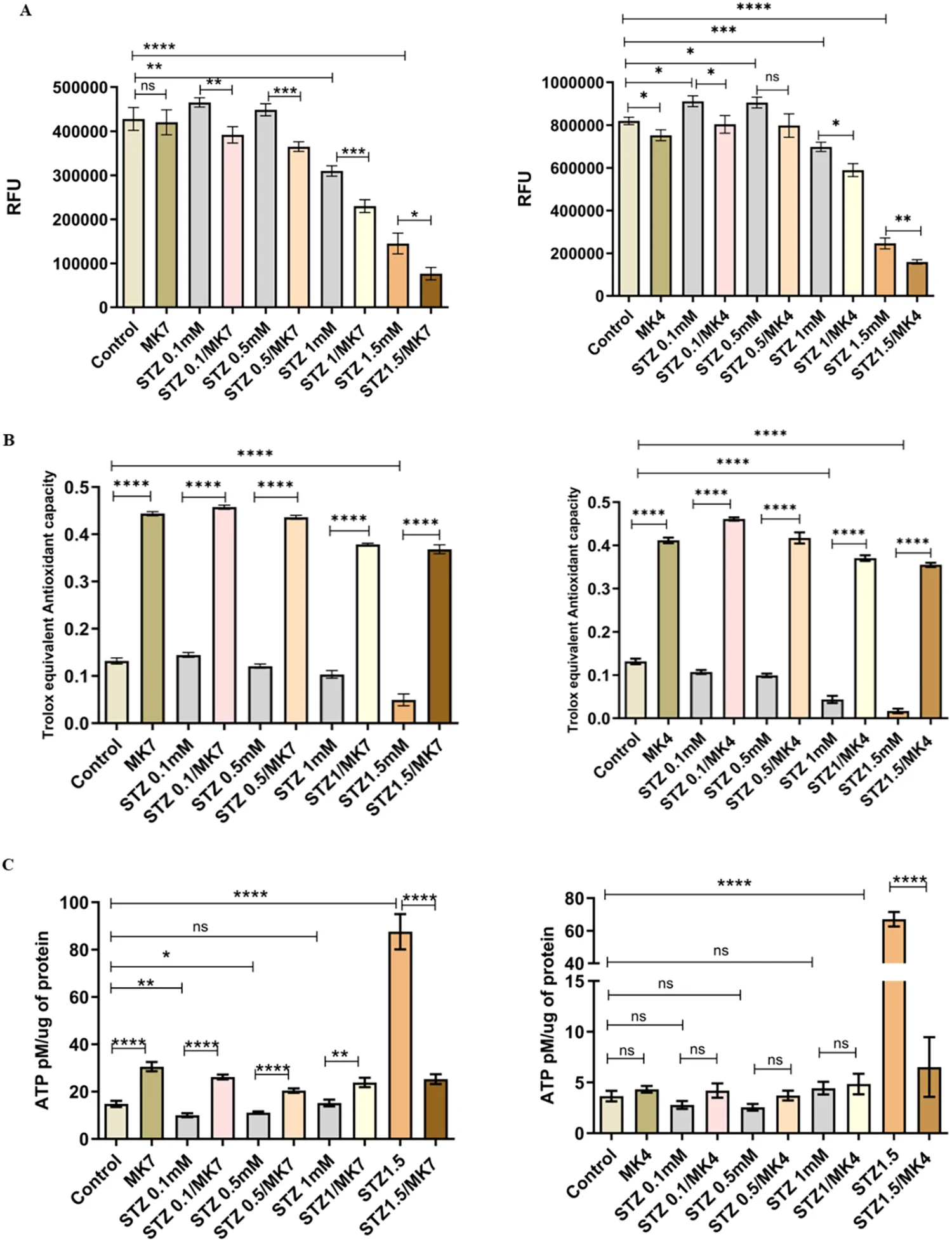
Effect of MK-4 and MK-7 on cellular homeostasis in d-SH-SY5Y cells exposed to STZ. (A) Intracellular reactive oxygen species (ROS) levels (*n* = 3). (B) Total antioxidant capacity (*n* = 3–4) and (C) intracellular ATP levels (*n* = 3–4) were measured following treatment. Data are presented as mean ± SEM. Statistical significance was determined by one-way ANOVA followed by Tukey’s post-hoc test; differences with *p*-values ≤ 0.05 were designated as significant. Brackets indicate the exact between-group comparisons, including untreated control versus STZ-only groups, untreated control versus MK-4/MK-7-only groups, and STZ-only groups versus corresponding MK-4- or MK-7-treated groups; ns, not significant; *p < 0.05, **p < 0.01, and ***p < 0.001 denote significant differences compared with the control and between the treatment groups. MK-7, menaquinone-7; MK-4, menaquinone-4; STZ, streptozotocin; STZ/MK-4, post-treatment of STZ-stressed cells with MK-4; STZ/MK-7, post-treatment of STZ-stressed cells with MK-7.

### 3.4 MK-4 and MK-7 Attenuate Streptozotocin-Induced DNA Damage

Streptozotocin acts as a strong alkylating agent that directly methylates DNA, resulting in strand breaks and cell death, while accumulating evidence also suggests that free radical-mediated oxidative stress by STZ plays a key role in its genotoxic and cytotoxic mechanisms.^60^ In this study, the genotoxic effect of STZ and the cytoprotective potential of dietary vitamin K2 isoforms (MK-4 and MK-7) were evaluated in d-SH-SY5Y cells using the neutral comet assay. Exposure of cells to STZ (0.5 and 1.5 mM) for 24 h resulted in a significant increase in DNA strand breaks, as indicated by an increase in % Tail DNA and olive tail moment (Figure 4A–C), compared with untreated control and MK-4- and MK-7-treated cells. Absolute values of Tail DNA % and olive tail moment for each treatment group are presented in Table 2. Our findings coincide with previous reports, which showed the STZ at 1 mM concentration resulted in O_2_^–^ radical-induced enhanced DNA fragmentation in isolated rat pancreatic β cells.^61^ Similarly, it was observed that STZ at 0.55 mM induced double-strand breaks in rat pancreatic islet cells.^62^ A recent report also showed that STZ injected in the rat brain resulted in DNA fragmentation, leading to AD-like pathological changes.^54^ In contrast, treatment of d-SH-SY5Y cells with MK-4 and MK-7 alone did not cause any DNA damage relative to that of untreated control cells, confirming the non-genotoxic nature of these two vitamin K2 isoforms (Figure 4A–C). This observation is consistent with previous safety evaluations showing that MK-7 lacks genotoxicity and systemic toxicity in preclinical studies, with a NOAEL of 4500 mg/kg/day reported in a 90-day oral toxicity study.^63^ For MK-4, however, EFSA identified 20 mg/kg bw/day as the LOAEL in a one-year rat study.^64^ Having established their genomic safety, the ability of MK-4 and MK-7 to counteract STZ-induced genotoxic stress was subsequently examined. Importantly, post-treatment of STZ (0.5 mM)-stressed cells with MK-4 and MK-7 for 24 h resulted in a marked reduction in DNA damage, as reflected by significant decreases in comet tail parameters compared with STZ treatment alone. Also, MK-7 post-treatment resulted in a significant reduction of DNA damage even at the maximum concentration of STZ (1.5 mM), as indicated by a greater reduction in comet tail parameters, in comparison to the post-treatment of MK-4 (Figure 4A–C). The protective effect of vitamin K2 against streptozotocin (STZ)-induced DNA damage in d-SH-SY5Y cells observed in this study is consistent with earlier reports demonstrating the efficacy of antioxidant interventions in mitigating STZ-mediated genotoxicity. Previous studies have shown that classical antioxidants, including ascorbic acid, trolox, and probucol, protect against STZ-induced DNA damage *in vivo*, thereby reducing tissue injury associated with hepatic and renal dysfunction.^65^ More recent investigations have further demonstrated that vitamins E, C, and D confer significant protection against STZ-induced DNA damage by attenuating oxidative stress, suppressing malondialdehyde (MDA) formation, and enhancing endogenous antioxidant defense systems in diabetic models.^66–68^ In addition, dietary vitamin B12 supplementation has been reported to alleviate type-1 diabetes-associated neuronal apoptosis, highlighting the broader neuroprotective potential of micronutrients under diabetic and oxidative stress conditions.^69^ In agreement with these findings, the present results demonstrate that vitamin K2 isoforms MK-4 and MK-7 effectively mitigate STZ-induced DNA damage in d-SH-SY5Y cells, with MK-7 exhibiting comparatively greater efficacy in preserving genomic integrity under oxidative stress. However, the current study does not determine whether this effect results from direct interference with STZ-mediated DNA alkylation or from reduced downstream oxidative stress.

**Figure 4:**
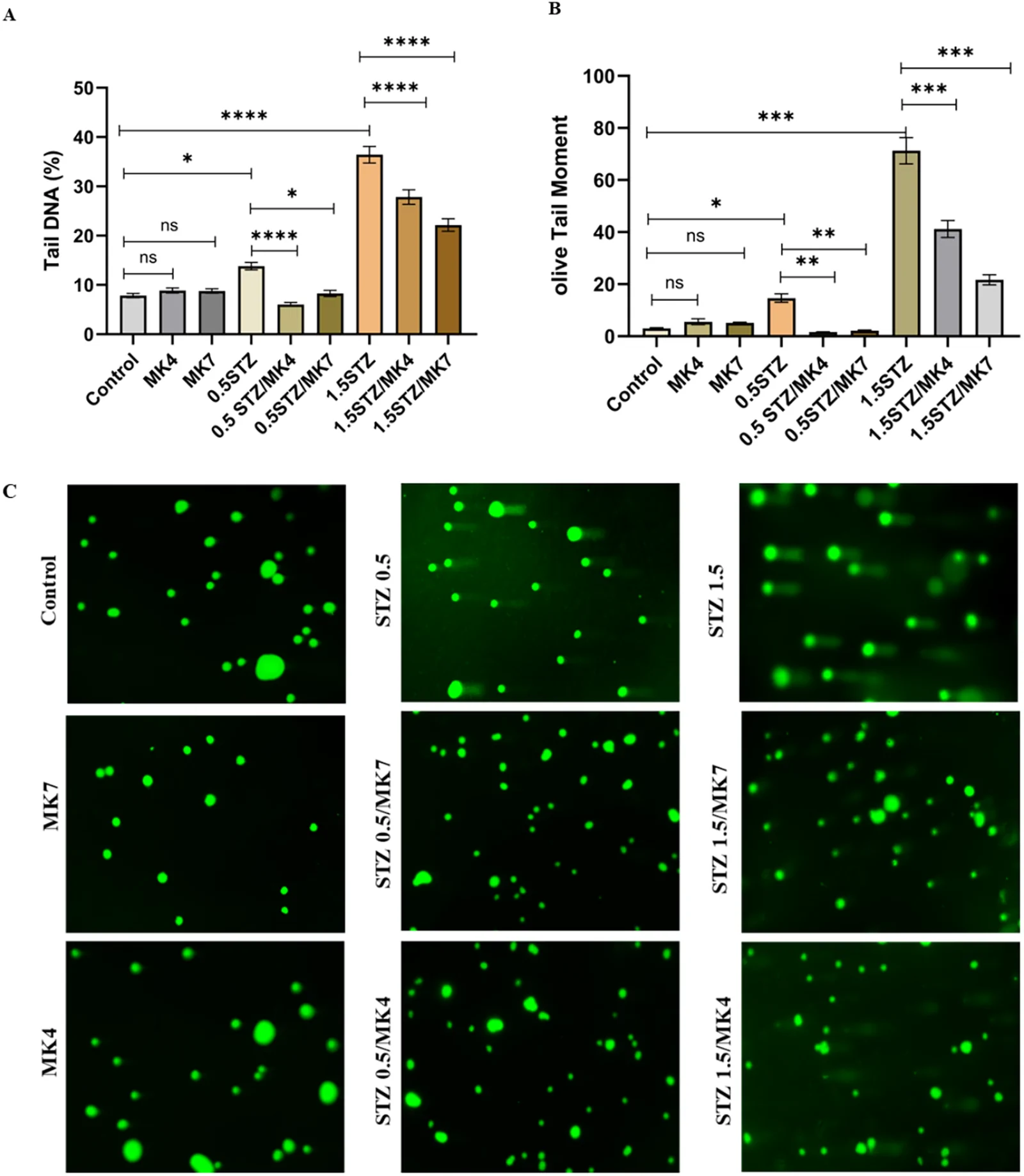
Effect of MK-4 and MK-7 on DNA damage induced by STZ in d-SH-SY5Y cells. DNA damage was assessed by a neutral comet assay. (A) Percentage of tail DNA and (B) olive tail moment was quantified to evaluate DNA strand breaks. (C) Representative fluorescence microscopy images of comets from each treatment group (scale bar: 100 μm). Data are presented as mean ± SEM. Statistical significance was determined by one-way ANOVA followed by Tukey’s post-hoc test; differences with *p*-values ≤ 0.05 were designated as significant. Brackets indicate the exact between-group comparisons, including untreated control versus STZ-only groups, untreated control versus MK-4/MK-7-only groups, and STZ-only groups versus corresponding MK-4- or MK-7-treated groups; ns, not significant; *p < 0.05, **p < 0.01, and ***p < 0.001 denote significant differences compared with the control and between the treatment groups. MK-7, menaquinone-7; MK-4, menaquinone-4; STZ, streptozotocin; STZ/MK-4, post-treatment of STZ-stressed cells with MK-4; STZ/MK-7, post-treatment of STZ-stressed cells with MK-7.

**Table 2:** Absolute Values of Tail DNA % and Olive Tail Moment in Different Treatment Groups Evaluated through CometScore 2.0

**treatment group**	**tail DNA %**	**olive tail moment**
**control**	7.837 ± 0.435	3.027 ± 0.229
**MK-4**	8.865 ± 0.512	5.558 ± 1.110
**MK-7**	8.771 ± 0.476	5.098 ± 0.311
**STZ 0.5**	13.82 ± 0.761	14.65 ± 1.606
**STZ0.5/MK-4**	6.037 ± 0.393	1.602 ± 0.112
**STZ0.5/MK-7**	8.267 ± 0.624	2.144 ± 0.250
**STZ 1.5**	36.42 ± 1.676	71.28 ± 5.056
**STZ 1.5/MK-4**	27.84 ± 1.476	41.22 ± 3.254
**STZ 1.5/MK-7**	22.16 ± 1.277	21.68 ± 1.979

### 3.5 MK-4 and MK-7 Prevent Mitochondrial Dysfunction in d-SH-Y5Y Cells Exposed to Streptozotocin

Mitochondria are essential regulators of cellular homeostasis, governing bioenergetic processes and apoptotic signaling pathways.^70^ Mitochondrial dysfunction, its altered intracellular distribution, and excessive mitochondrial fragmentation are widely recognized as characteristic features of neurodegenerative progression in Alzheimer’s disease.^71^ Given that streptozotocin induces oxidative stress-mediated mitochondrial dysfunction^3,6^ and that mitochondria serve as a primary intracellular source of ROS,^70^ we evaluated mitochondrial health in d-SH-SY5Y cells following exposure to the highest concentration of STZ (1.5 mM) by assessing mitochondrial ROS generation, mitochondrial morphology, and mitophagy, in order to determine the Mito-protective potential of dietary vitamin K2 isoforms (MK-4 and MK-7) under severe stress conditions. Excessive mt-ROS was detected in d-SH-SY5Y cells treated with STZ (1.5 mM), as indicated by the striking increase in MitoSOX/Biotracker signal ratio in comparison to that of untreated control cells (Figure 5A1,A2). These results were consistent with our previous study where STZ treatment of undifferentiated SH-SY5Y cells resulted in a decrease of mitochondrial membrane potential in a dose-dependent manner.^10^ Since mitochondrial structural integrity determines functional efficiency and resilience, alterations in mitochondrial function are closely associated with changes in morphology.^72^ Changes in mitochondrial morphology, analyzed as mitochondrial footprints, were found to be significantly reduced, with mitochondria appearing fragmented and less dense, when cells were exposed to STZ at 1.5 mM (Figure 5B). In contrast, post-treatment of STZ-exposed cells with MK-4 and MK-7 led to a significant reduction of MitoSOX-to-Biotracker signal ratio (Figure 5A), along with a significant increase in mitochondrial footprint in comparison to STZ-treated cells, with MK-7 demonstrating slightly greater efficacy compared with MK-4 (Figure 5B). Also, d-SH-SY5Y cells treated with MK-4 and MK-7 alone showed a similar elongated mitochondrial network in the cellular region and neurites as in untreated control cells (Figure 5B). This indicates that MK-4 and MK-7 not only balanced mitochondrial redox status but also restored mitochondrial area to levels similar to those of untreated controls and cells treated with MK-4 and MK-7 alone (Figure 5B).

**Figure 5:**
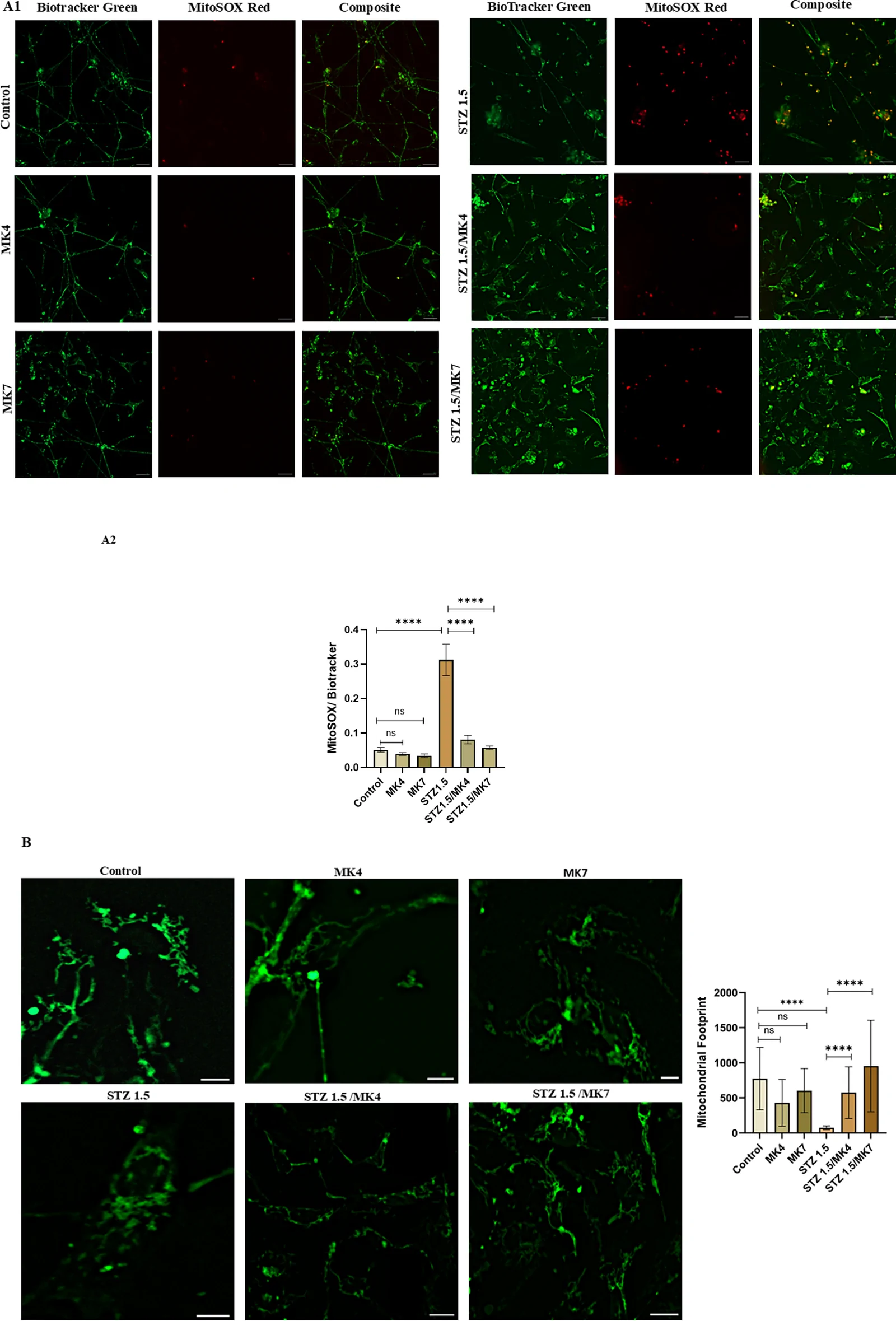

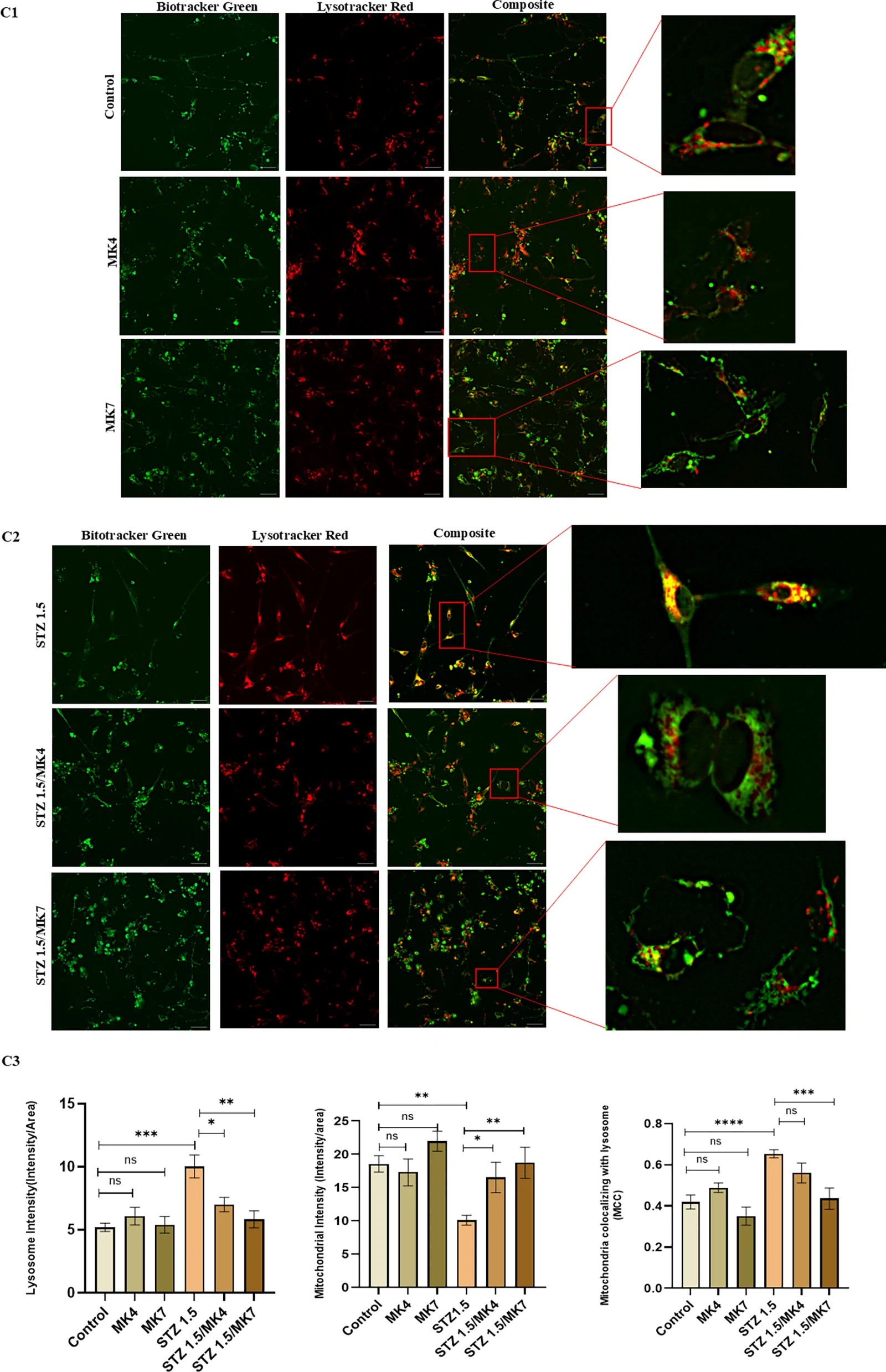
MK-4 and MK-7 attenuate mitochondrial damage induced by STZ in d-SH-SY5Y cells. (A) Mitochondrial reactive oxygen species (ROS) generation. (A1) Fluorescence microscopy images show MitoSOX Red (mt-ROS) and mitochondrial mass visualized with Biotracker Green for each treatment group (scale bar: 100 μm). (A2) The bar graph represents the MitoSOX-to-Biotracker signal ratio (*n* = 3). (B) Mitochondrial morphology was assessed by fluorescence microscopy using Biotracker Green and quantified as mitochondrial footprint using the miNA plugin of ImageJ software (scale bar: 100 μm). (C1, C2) Mitophagy was evaluated by mitochondrial-lysosomal colocalization analysis, where fluorescence microscopy images represent mitochondria stained using Biotracker Green and lysosome using Lysotracker Red. Colocalization was visualized as yellow (red + green) in composite images and was quantified using the JACoP plugin in ImageJ software (scale bar: 100 μm). (C3) Manders’ colocalization coefficient M2 represents the fraction of mitochondrial signals (Biotracker Green) colocalizing with lysosomal signals (Lysotracker Red). Data are presented as mean ± SEM. Statistical significance was determined by one-way ANOVA followed by Tukey’s post-hoc test; differences with *p*-values ≤ 0.05 were designated as significant. Brackets indicate the exact between-group comparisons, including untreated control versus STZ-only groups, untreated control versus MK-4/MK-7-only groups, and STZ-only groups versus corresponding MK-4- or MK-7-treated groups; ns, not significant; *p < 0.05, **p < 0.01, and ***p < 0.001 denote significant differences compared with the control and between the treatment groups. MK-7, menaquinone-7; MK-4, menaquinone-4; STZ, streptozotocin; STZ/MK-4, post-treatment of STZ-stressed cells with MK-4; STZ/MK-7, post-treatment of STZ-stressed cells with MK-7.

Oxidative stress promotes mitochondrial fragmentation and activation of mitochondria-dependent apoptotic pathways.^73^ As a key intracellular signaling mediator, oxidative stress can also trigger autophagy, and selective removal of damaged mitochondria via mitophagy is essential for preserving cellular homeostasis.^74^ Consistent with enhanced mitochondrial ROS and fragmentation, STZ significantly increased mitophagy, as evidenced by elevated mitochondrial-lysosomal colocalization (Manders’ M2 coefficient; Table 3) and increased lysosomal intensity relative to mitochondrial signal (Figure 5C1,C2). However, excessive mitophagy under oxidative stress conditions has been reported to contribute to neuronal injury when mitochondrial clearance exceeds biogenesis capacity.^75,76^ In contrast, post-treatment with MK-4 and MK-7 significantly reduced mitochondrial-lysosomal colocalization (Manders’ M2 coefficient; Table 3) and normalized mitochondrial intensity ratios in comparison to lysosomal intensity (Figure 5C3), indicating restoration of mitochondrial quality control mechanisms. In line with our results, a previous report also showed that MK-7 protected astrocytes from hypoxia-induced cytotoxicity by inhibiting mitochondrial dysfunction and inflammatory response.^56^ Vitamin K2 has been reported to attenuate apoptosis in PC12 cells, partly through activation of the p38 MAP kinase signaling pathway.^37^ In a Drosophila model expressing arctic mutant Aβ42, vitamin K2 reduced β-amyloid accumulation, improved ATP production, restored mitochondrial function, enhanced locomotor performance, and increased lifespan.^77^ Furthermore, vitamin K2 was shown to alleviate sevoflurane-induced tau hyperphosphorylation and associated cognitive deficits in neonatal mice.^78^ Similarly, in SH-SY5Y cells, MK-7 has also been demonstrated to counteract 6-hydroxydopamine-induced mitochondrial dysfunction by regulating mitochondrial quality control mechanisms.^11^ These findings highlight the substantial neuroprotective potential of vitamin K2, largely attributed to its ability to preserve mitochondrial integrity and function. Apart from neuronal cells, vitamin K2 was found to attenuate mitochondrial damage in renal proximal tubular cells^40^ and in LPS-induced ALI in mice.^79^ Taken together, our results showed that dietary vitamin K2 isoforms (MK-4 and MK-7) mitigate STZ-induced mitochondrial dysfunction by reducing mt-ROS, preserving mitochondrial network integrity and normalizing mitophagy. The enhanced efficacy of MK-7 suggests isoform-specific differences in mitochondrial protection. Restoration of mitochondrial structural and functional integrity likely underlies the broader neuroprotective effects observed under STZ-induced stress conditions.

**Table 3:** Effect of MK-4 and MK-7 on Mitochondrial Parameters in STZ-Treated d-SH-SY5Y Cells

**treatment group**	**MitoSOX/mitochondria ratio**	**mitochondrial footprint (μm** ^**2**^ **)**	**Manders’ coefficient (M2)**
**control**	0.051 ± 0.006	774.4 ± 96.75	0.418 ± 0.033
**MK-4**	0.038 ± 0.004	429.3 ± 65.32	0.488 ± 0.023
**MK-7**	0.033 ± 0.005	602.4 ± 74.37	0.350 ± 0.044
**STZ 1.5**	0.312 ± 0.045	73.42 ± 4.196	0.654 ± 0.019
**STZ 1.5/MK-4**	0.081 ± 0.012	575.5 ± 78.37	0.560 ± 0.047
**STZ 1.5/MK-7**	0.057 ± 0.005	880.6 ± 92.66	0.436 ± 0.051

### 3.6 MK-4 and MK-7 Attenuate Streptozotocin-Induced ER-Golgi Disorganization

Chronic oxidative stress-mediated mitochondrial dysfunction is known to promote the accumulation of unfolded and misfolded proteins within the ER, thereby triggering ER stress and activation of the unfolded protein response (UPR). The UPR represents an adaptive cellular mechanism that facilitates the removal of misfolded proteins while simultaneously upregulating antioxidant and cytoprotective pathways to support cell survival under stress conditions.^4,80^ In neuronal systems, prolonged or unresolved ER stress is closely linked to structural remodeling of the secretory pathway and initiation of cell death signaling.^81^ Given the close morphological and functional coupling between the ER and Golgi apparatus, disruption of one compartment frequently impacts the other, making it difficult to distinguish ER versus Golgi-initiated pathways of cellular injury. Indeed, pharmacological induction of ER stress using agents such as thapsigargin has been shown to cause Golgi dispersal and fragmentation, reflecting impaired ER/Golgi trafficking.^82^ Similarly, oxidative stressors and excitotoxic insults, including NMDA and nitric oxide donors, have been reported to perturb ER function, leading secondarily to Golgi fragmentation.^83^ In the present study, exposure of d-SH-SY5Y cells to a high dose of STZ resulted in a marked reduction in Golgi/ER colocalization. Intensity-based quantification of fluorescence microscopy images revealed a significant reduction in Golgi- and ER-specific fluorescence intensities following STZ treatment, indicating stress-induced alterations in these organelles. This reduction was accompanied by a marked decrease in Golgi/ER colocalization, visualized as reduced yellow puncta in merged images. Quantitative colocalization analysis using the JACoP plugin demonstrated a significant decrease in Manders’ overlap coefficient (M2), indicating reduced Golgi signal overlapping with the ER and reflecting pronounced changes in ER/Golgi spatial organization under STZ-induced stress (Figure 6). Absolute values of Manders’ coefficient is shown in Table 4. Emerging evidence further suggests that the Golgi apparatus itself can actively participate in stress-induced cell death signaling. Under oxidative stress conditions, the cell-death-related protein annexin II undergoes phosphorylation by the Src family kinase Lyn, followed by translocation from the Golgi to the ER, implicating the Golgi as an upstream regulator of ER-mediated stress responses.^81,83^ Such findings support the notion that Golgi dysfunction observed in the present study may not merely be a downstream consequence of ER stress, but rather an active contributor to stress signaling cascades.^84^ Disruption of Golgi/ER colocalization is a recognized pathological feature of neurodegeneration and is largely driven by Golgi fragmentation and breakdown of both anterograde (ER-to-Golgi) and retrograde (Golgi-to-ER) trafficking.^85,86^ Consistent with this, DNA damage itself has been shown to induce Golgi dispersal independent of apoptosis. Specifically, genotoxic stress activates DNA-dependent protein kinase (DNA-PK), which phosphorylates the Golgi protein GOLPH3, enhancing its interaction with the actin cytoskeleton via MYO18A and promoting Golgi fragmentation.^7,87^ Impairment of either anterograde or retrograde trafficking is sufficient to destabilize ER/Golgi communication, ultimately collapsing secretory pathway organization and triggering cellular stress responses that lead to injury.^88,89^ Thus, preservation of ER/Golgi trafficking integrity is essential for maintaining Golgi morphology and cellular homeostasis. In this context, the ability of vitamin K2 isoforms MK-4 and MK-7 to restore Golgi and ER fluorescence intensity and to normalize Golgi/ER colocalization (normalized M2 values) following STZ exposure suggests effective mitigation of stress-induced disruption of the secretory pathway, as shown in Figure 6 and Table 4. The more pronounced restorative effect observed with MK-7 further supports isoform-specific differences in cytoprotective efficacy (Figure 6). Results from this study coincide with our previous study where MK-4 has been found to attenuate chronic ER stress induced by STZ and menadione by decreasing the expression of the ER stress marker CHOP and reducing the activation of key unfolded protein response pathways, indicated by a reduction in the expression of p-IRE1α and GSK-3β, thus supporting its role in improving ER homeostasis under oxidative stress conditions.^10^ Thus, vitamin K2 ameliorated neuronal cell death by inhibiting chronic ER stress-mediated signaling, highlighting its potential in preserving organelle integrity beyond mitochondrial protection. Overall, these findings indicate that vitamin K2 alleviates STZ-induced ER/Golgi disorganization, thereby contributing to improved cellular homeostasis and neuronal stress resilience.

**Figure 6:**
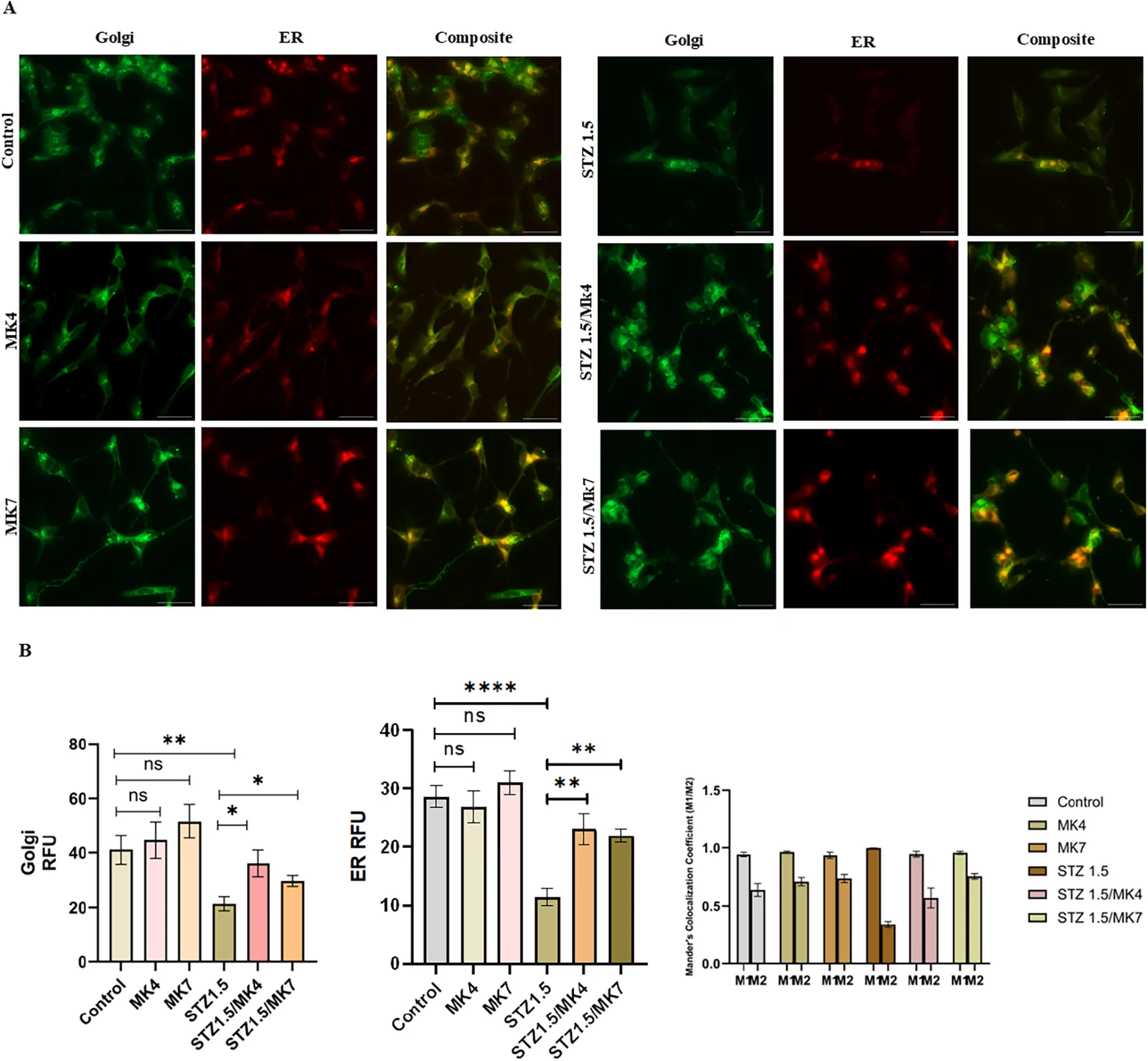
Effect of MK-4 and MK-7 on STZ-induced endoplasmic reticulum and Golgi stress in d-SH-SY5Y cells. (A) Fluorescence microscopy images representing Golgi (green) and endoplasmic reticulum (red) when d-SH-SY5Y cells were stained using Cytopainter-Golgi/ER staining kit. Composite images show the colocalization of Golgi and ER visualized as yellow (red + green) (scale bar: 100 μm). (B) Bar graphs represent quantitative assessment of fluorescence intensities (Golgi; endoplasmic reticulum) and of colocalization using the JACoP plugin in ImageJ software, where M1 represents the fraction of ER (red) signal colocalizing with Golgi (green), and M2 represents the fraction of Golgi (green) signal colocalizing with ER (red). Data are presented as mean ± SEM. Statistical significance was determined by one-way ANOVA followed by Tukey’s post-hoc test; differences with *p*-values ≤ 0.05 were designated as significant. Brackets indicate the exact between-group comparisons, including untreated control versus STZ-only groups, untreated control versus MK-4/MK-7 only groups, and STZ-only groups versus corresponding MK-4- or MK-7-treated groups; ns, not significant; *p < 0.05, **p < 0.01, and ***p < 0.001 denote significant differences compared with the control and between the treatment groups. MK-7, menaquinone-7; MK-4, menaquinone-4; STZ, streptozotocin; STZ/MK-4, post-treatment of STZ-stressed cells with MK-4; STZ/MK-7, post-treatment of STZ-stressed cells with MK-7.

**Table 4:** Manders’ Colocalization Coefficient M2, Representing Fraction of Golgi Signal Overlapping with Endoplasmic Reticulum Signal

**treatment group**	**MCC (M2) Golgi colocalization with ER**
**control**	0.638 ± 0.055
**MK-4**	0.709 ± 0.035
**MK-7**	0.737 ± 0.035
**STZ 1.5**	0.339 ± 0.026
**STZ 1.5/MK-4**	0.569 ± 0.084
**STZ 1.5/MK-7**	0.755 ± 0.024

### 3.7 MK-4 and MK-7 Promote Neurite Outgrowth in Stressed Cells

Neurite outgrowth was assessed in d-SH-SY5Y cells to evaluate the effect of vitamin K2 isoforms (MK-4 and MK-7) on neuronal morphology under STZ-induced stress. Quantitative analysis of phase-contrast images using NeuronJ revealed that STZ exposure at a high dose resulted in a marked reduction in mean neurite length and complexity compared with untreated control cells, indicating impaired neuronal differentiation and structural integrity (Figure 7). Absolute values of neurite growth in μm in respective treatment groups are shown in Table 5. These findings are consistent with previous reports of *in vitro* and *in vivo* studies showing that STZ induces oxidative stress, mitochondrial dysfunction, and impaired insulin signaling, resulting in altered neuronal morphology and degeneration.^3,54,90–92^ Whereas post-treatment of STZ-stressed cells with MK-4 or MK-7 (50 μM, 24 h) significantly enhanced neurite extension relative to STZ alone, indicating partial restoration of neuronal structural integrity. Both isoforms increased mean neurite length, suggesting recovery of neuritogenic capacity under stress conditions (Figure 7 and Table 5). The restoration of neurite outgrowth by vitamin K2 aligns with prior studies showing that vitamin K compounds promote neuronal differentiation and neuritogenesis. First, Tsang and Kamei (2002) reported that vitamins K1 and K2 enhanced nerve growth factor (NGF)-induced neurite outgrowth in PC12D cells via modulation of intracellular signaling pathways. Beyond its antioxidant properties, vitamin K has been implicated in sphingolipid metabolism, a critical determinant of neurite formation and membrane architecture. Sphingolipids, including ceramide and sphingomyelin, regulate membrane microdomain organization, axonal elongation, and growth cone dynamics^12,93^ MK-4 is the predominant form of vitamin K in the brain and has been shown to influence enzymes involved in sphingolipid biosynthesis.^8^ Disruption of sphingolipid homeostasis impairs neurite extension, whereas restoration of sphingolipid balance promotes neuronal differentiation and process elongation.^93^ In the context of other vitamins promoting neurite outgrowth, it has been observed that a combination of vitamins B1, B6, and B12 acted in biochemical synergy and promoted neurite growth of healthy neurons in comparison to individually administered B vitamins within the NG108-15 cell model.^24^ Similarly, vitamin D increased neurite length and velocity in the first 12 h of the developmental phase in rat cortical neurons.^94^ Recently, it was observed that synthetically designed vitamin K homologue (Compound 7) was found to effectively cross the BBB and convert into MK-4 over time, and was found to have a strong neuronal differentiation activity in mouse neuronal progenitor cells.^13^ In the present study, among the two isoforms, MK-7 produced a more pronounced increase in neurite length relative to MK-4 (Figure 7). Treatment with MK-4 or MK-7 alone did not adversely affect neurite morphology and maintained neurite length comparable to control cells. The observed recovery of neurite outgrowth in vitamin K2-treated cells suggests improved neuronal health and structural stability following STZ-induced stress. Since neurite extension is a key indicator of differentiation and functional integrity, these findings support a role for MK-4 and MK-7 in maintaining neuronal architecture under metabolic stress. However, certain limitations of the present study should be noted. Although the study provides an integrated phenotypic assessment of MK-4- and MK-7-associated protection against STZ-induced injury, it does not directly define the upstream signaling pathways involved. Further, as the experiments were carried out using an *in vitro-*differentiated SH-SY5Y cell model, additional validation in complementary neuronal models and *in vivo* systems will be required to support broader translational relevance. In conclusion, findings of this study promote the understanding of the cytoprotective potential of MK-4 and MK-7 under STZ-induced oxidative stress, DNA damage, and compromised neurite integrity and support the potential relevance of dietary vitamin K2 isoforms as a nutritional strategy for preventing neurodegenerative processes.

**Figure 7:**
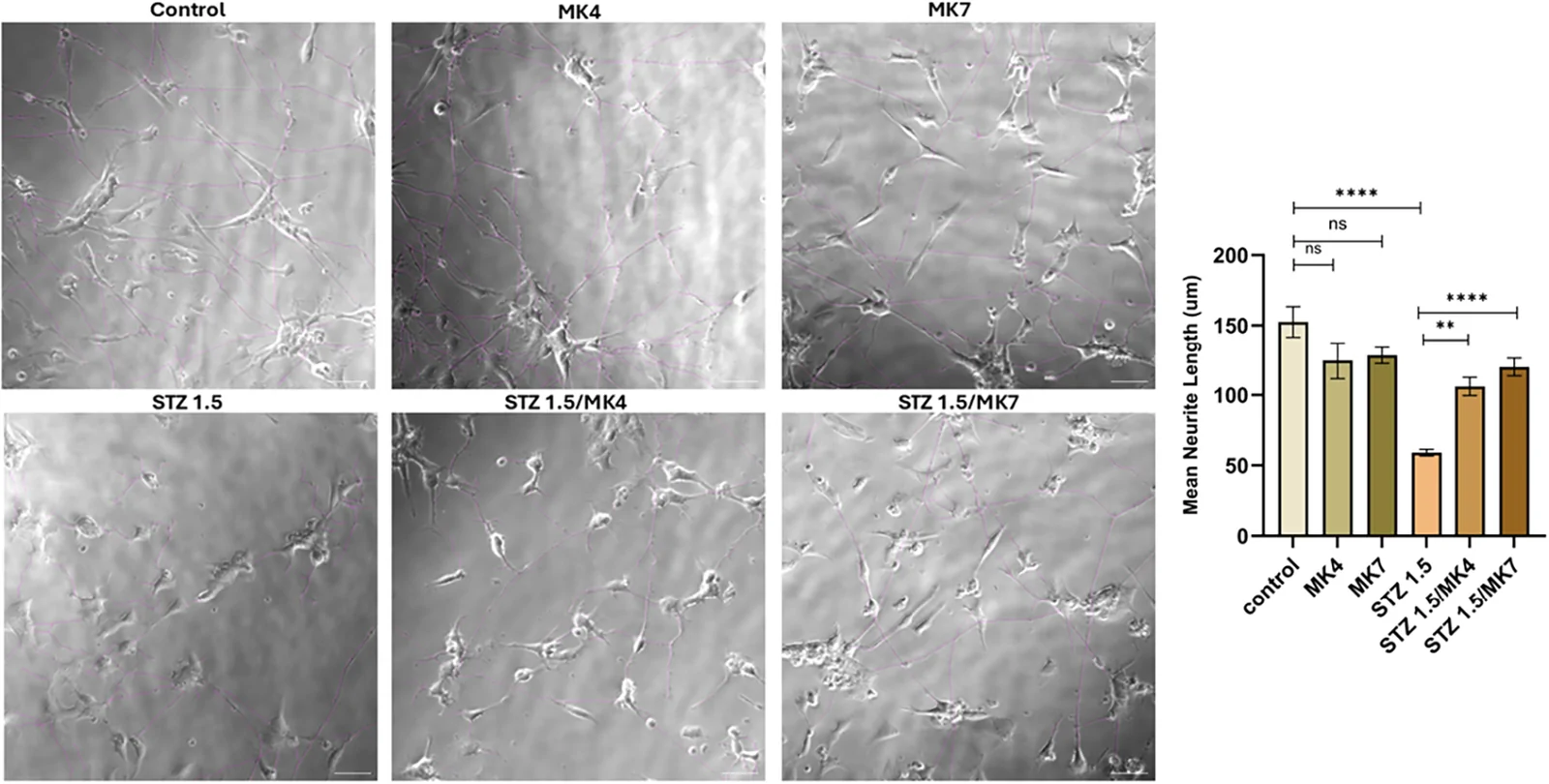
MK-4 and MK-7 promote neurite outgrowth in STZ-induced stress in d-SH-SY5Y cells. Right: Phase-contrast images with neurites traced using the NeuronJ plugin of ImageJ software (scale bar: 100 μm). Left: Bar graph represents quantitative assessment of neurite length in μm using NeuronJ plugin (*n* = 3–4). Data are presented as mean ± SEM. Statistical significance was determined by one-way ANOVA followed by Tukey’s post-hoc test; differences with *p*-values ≤ 0.05 were designated as significant. Brackets indicate the exact between-group comparisons, including untreated control versus STZ-only groups, untreated control versus MK-4/MK-7-only groups, and STZ-only groups versus corresponding MK-4- or MK-7-treated groups; ns, not significant; *p < 0.05, **p < 0.01, and ***p < 0.001 denote significant differences compared with the control and between the treatment groups. MK-7, menaquinone-7; MK-4, menaquinone-4; STZ, streptozotocin; STZ/MK-4, post-treatment of STZ-stressed cells with MK-4; STZ/MK-7, post-treatment of STZ-stressed cells with MK-7.

**Table 5:** Absolute Values of Neurite Growth in μM in Different Treatment Groups Evaluated Using the NeuronJ Plugin of ImageJ

**treatment group**	**mean neurite growth** (μm)
**control**	152.5 ± 10.87
**MK-4**	124.6 ± 12.93
**MK-7**	128.2 ± 5.734
**STZ 1.5**	59.07 ± 2.328
**STZ1.5/MK-4**	106.1 ± 6.450
**STZ1.5/MK-7**	119.9 ± 6.354

## Data Availability

Data will be made available upon request.

## Abbreviations

AD: Alzheimer’s disease
aMCI: amnestic mild cognitive impairment
ATP: adenosine triphosphate
BDNF: brain-derived neurotropic factor
CAT: catalase
COQ10: coenzyme Q10
DMEM: Dulbecco’s modified Eagle’s medium
DNA: deoxyribonucleic acid
d-SH-SY5Y: differentiated SH-SY5Y
ER: endoplasmic reticulum
FBS: fetal bovine serum
GAP-43: growth-associated protein 43
H_2_O_2_: hydrogen peroxide
HBSS: Hank’s balanced salt solution
LDH: lactate dehydrogenase
LOAEL: lowest-observed-adverse-effect level
MitoSOX: mitochondrial superoxide
MK-4: menaquinone-4
MK-7: menaquinone-7
NOAEL: no-observed-adverse-effect level
NSE: neuron-specific enolase
PINK1: PTEN-induced kinase 1
RA: retinoic acid
ROI: region of interest
ROS: reactive oxygen species
SOD: super oxide dismutase
STZ: streptozotocin
SV2: synaptic vesicle protein 2
SYN: synaptophysin
TROLOX: 6-hydroxy-2,5,7,8-tetramethylchroman-2-carboxylic acid
